# Expression of toll-like receptor 4 in maternal monocytes of patients with gestational diabetes mellitus

**DOI:** 10.3892/etm.2013.1360

**Published:** 2013-10-24

**Authors:** BAO-GUO XIE, SONG JIN, WEI-JIE ZHU

**Affiliations:** 1 Department of Developmental and Regenerative Biology, College of Life Science and Technology, Jinan University, Guangzhou, Guangdong 510632, P.R. China; 2 Department of Obstetrics and Gynecology, Affiliated Hospital of Hainan Medical College, Haikou, Hainan 570102, P.R. China

**Keywords:** gestational diabetes mellitus, toll-like receptor, tumor necrosis factor-α, monocyte

## Abstract

Toll-like receptors (TLRs) are pattern recognition receptors and play an important role in innate immune responses and the occurrence of inflammatory disease. TLR4 is a member of the TLR family and its activation is capable of inducing inflammatory responses, reflecting a relationship between the innate and adaptive immune systems. However, whether TLR4 is expressed in patients with gestational diabetes mellitus (GDM) has not been elucidated. The aim of the present study was to investigate whether TLR4 is expressed in maternal peripheral blood monocytes of patients with GDM. A case-control study, using standard quantitative polymerase chain reaction and western blotting, was performed to assess the TLR4 expression in 30 females with GDM and 32 healthy pregnant females at similar gestational ages. Serum tumor necrosis factor (TNF)-α levels were assessed using ELISA in all the females. The TLR4 expression levels in the maternal peripheral blood monocytes and the serum TNF-α levels were increased in females with GDM compared with healthy pregnant females (P<0.05). Additionally, there was a positive correlation between the TLR4 expression level in peripheral blood monocytes and serum TNF-α levels in all the females. These results indicate that TLR4-mediated release of inflammatory cytokines may represent one factor leading to increased glucose levels in patients with GDM. In addition, TLR4 may be involved in the pathogenesis of GDM.

## Introduction

Gestational diabetes mellitus (GDM) is defined as a glucose intolerance of varying severity with an onset or first diagnosis during pregnancy (1). GDM occurs in 4–10% of pregnancies and is associated with maternal and fetal complications, as well as long-term consequences, including metabolic syndrome, type 2 diabetes mellitus (T2DM) and cardiovascular disease (2,3). Although the pathogenesis of GDM has not been completely elucidated, the innate immune system has been reported to be involved (4). In addition, several types of cytokines have been shown to have the ability to interfere with insulin signaling, as well as the development of insulin resistance (IR), in patients with GDM (5).

Toll-like receptors (TLRs) recognize preserved patterns and are important in the regulation of innate immune responses and inflammation (6). TLRs are expressed in numerous types of cells, including adipose cells and monocytes. These cells are the predominant cells of the innate immune system and are pivotal in diabetes (7). Among the TLRs, TLR4 is a particularly important receptor. TLR4 is the receptor for lipopolysaccharide (LPS) from Gram-negative bacteria, and affects the innate immune response, the prevalence of T2DM and the metabolic system (8). TLR4 expression has been identified in numerous cells and tissues, primarily in monocytes (9). TLR4 is important for the regulation of the immune response and inflammatory reaction (10). In addition, TLR4 induces the production of proinflammatory cytokines, leading to an impairment of tissue-specific effects (11,12). However, whether TLR4 is expressed in maternal monocytes of patients with GDM has not been evaluated.

The current study was conducted to investigate whether TLR4 is expressed in maternal monocytes of patients with GDM and to elucidate the roles of TLR4 in the pathogenesis of GDM. Novel insights into the involvement of TLR4 in the pathogenesis of GDM may provide an opportunity to trace the underlying pathogenesis of GDM and, if proven, may be conducive to improving the treatment of the disease.

## Materials and methods

### Subjects

A total of 62 females (21–39 years old) at ≥37 weeks of gestation were involved in this study, including 32 females with GDM and 30 healthy pregnant females. Females with GDM were selected by a screening and diagnostic program according to the criteria of the Fourth Workshop Conference of Gestational Diabetes (13). Females with multiple pregnancies, fetal anomalies, preexisting hypertension or DM, or chronic disease were excluded. All females with GDM were treated with insulin and IR was estimated using the homoeostasis model assessment (HOMA)-IR. The protocol was approved by the local Ethics Committee of College of life and Technology, Jinan University (Guangzhou, China) and written informed consent was obtained from all females.

### Isolation of monocytes

Maternal peripheral blood was collected using tubes treated with EDTA and the peripheral blood monocytes were isolated by density gradient centrifugation using Ficoll-Paque PLUS (Amersham, Piscataway, NJ, USA). The negative isolation of human monocytes was performed using a Monocyte Isolation kit II (Miltenyi Biotech, Bergisch Gladbach, Germany), in accordance with the manufacturer’s instructions. Following centrifugation at 400 × g for 30 min, the cells were washed with phosphate-buffered saline at 4°C. The viabilities of monocytes at >90% confluence were calculated using 0.4% trypan blue. The monocytes were stored at −80°C until use.

### RNA isolation and quantitative polymerase chain reaction (qPCR)

Total RNA from the monocytes was isolated using a commercial kit (Omega Bio-Tek, Norcross, GA, USA), in accordance with the manufacturer’s instructions. The RNA concentration and purity were determined using 1.0% agarose gel electrophoresis with an optical density 260/280 absorption ratio of >1.8. cDNA was synthesized in a 20-μl reaction mixture containing 2 μg total RNA, using the Omniscript reverse transcription kit (Takara Bio, Inc., Otsu, Japan) and oligo(dT) primers, following the manufacturer’s instructions. TLR4 mRNA expression was measured with the ABI PRISM 7300 Sequence Detection System using the SYBR^®^ Green PCR Master mix (Applied Biosystems, Foster City, CA, USA). The following primers were used for analysis: TLR4 mRNA forward, 5′-AGTGTGTGTGTCCGCATGAT-3′ and reverse, 5′-CCACTTGGGGTCTAAGAACG-3′; 18S rRNA forward, 5′-TTCGGAACTGAGGCCATGAT-3′ and reverse, 5′-CGAACCTCCGACTTTCGTTT-3′. qPCR was performed with a 20-μl reaction mixture, using the SYBR Premix *Ex Taq*™ II kit (Takara Bio, Inc.), according to the manufacturer’s instructions. The PCR profile was obtained as follows: Initial activation step at 95°C for 5 min, followed by 40 cycles of denaturation, annealing and amplification (95°C for 15 sec, 60°C for 30 sec and 72°C for 15 sec, respectively). With regard to the internal control, the expression of the house-keeping gene, 18S rRNA, was examined under the same reaction conditions. The experiment was repeated in triplicate. Following amplification, melting curve analysis was conducted for the product formed.

### Western blotting

Western blotting was performed using the same monocytes as qPCR. Total protein was extracted from the monocytes using a cell lysis buffer and protease inhibitor cocktail. Following centrifugation at 10,000 × g and 4°C for 15 min, the protein concentration was assessed using the Bradford protein assay kit (Bio-Rad Laboratories, Hercules, CA, USA). The protein samples (20 μg) were loaded onto a 12% SDS-PAGE gel and transferred onto polyvinylidene difluoride (PVDF) membranes (Amersham Biosciences, Piscataway, NJ, USA). The membranes were blocked using 5% non-fat dry milk in a Tris-buffered sodium chloride-Tween-20 (TBST) solution (20 mmol/l Tris, pH 7.6, 137 mmol/l sodium chloride and 0.1% Tween-20) at room temperature for 1 h. The PVDF membranes were subsequently incubated with monoclonal antibodies against human TLR4 (1:1,000; Abcam, Cambridge, MA, USA) overnight at 4°C. Following this, the membranes were incubated with secondary antibody goat anti-rabbit horseradish peroxidase conjugate (1:2,000; Abcam) at room temperature for 2 h. Following three 10-min washes in TBST, the immunoreactive bands were detected using western blotting chemiluminescence luminol reagents (Santa Cruz Biotechnology, Inc., Santa Cruz CA, USA). The band intensities were quantified using scanning densitometry (Bio-Rad Quantity One software; Bio-Rad).

### Serum tumor necrosis factor (TNF)-α level analysis

The maternal peripheral blood samples were collected and transferred to centrifuge tubes. The blood samples were centrifuged at 3,000 × g for 15 min to separate the plasma and maintained at −80°C until analysis. Serum TNF-α levels were measured using a commercial TNF-α ELISA kit (BioSource International, Inc., Camarillo, CA, USA), in accordance with the manufacturer’s instructions.

### Statistical analysis

All experimental data are expressed as the mean ± standard deviation. Statistical analyses were performed with SPSS 17.0 statistical software (SPSS, Inc., Chicago, IL, USA). Inter-group differences were compared using the Student’s t-test and the correlation analyses were conducted using Pearson’s linear correlation analysis. P<0.05 was considered to indicate a statistically significant difference.

## Results

### Patient characteristics

The characteristics of the studied patients are listed in Table I. There were no significant differences in age, body mass index, blood pressure, gestational age or birth weight between the normal control and GDM groups (P>0.05). However, the blood glucose levels and HOMA-IR score were significantly higher in the GDM group than in the normal control group (P<0.05).

### qPCR and western blotting

**Figure 1:**
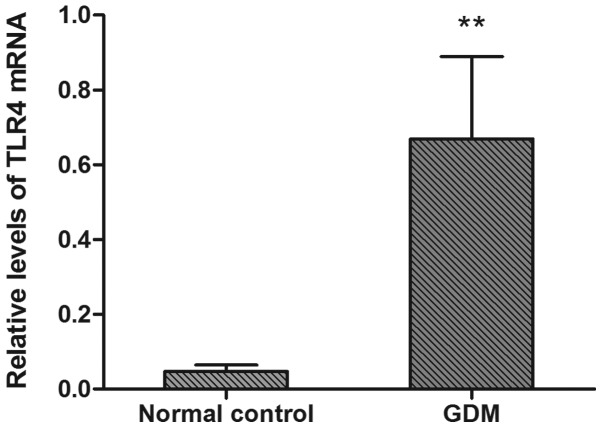
Relative levels of TLR4 mRNA were normalized to β-actin mRNA. Data are presented as the mean ± standard deviation. TLR4 mRNA levels were increased in females with GDM compared with the normal control females. ^**^ P<0.05, vs. normal controls. TLR4, toll-like receptor 4; GDM, gestational diabetes mellitus.

The TLR4 mRNA expression results are shown in Fig. 1. TLR4 mRNA expression levels were significantly higher in the GDM group than in the normal control group (P<0.05).

**Figure 2:**
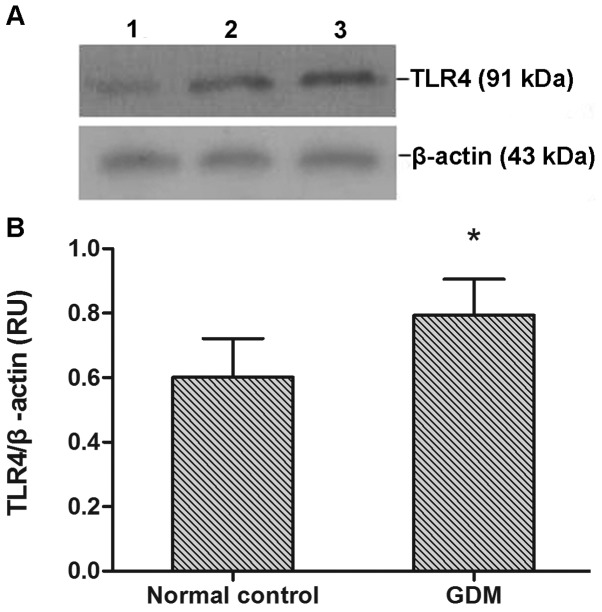
Expression of TLR4 protein in monocytes. (A) TLR4 protein and β-actin were detected. The intensity of TLR4 protein is shown in the maternal monocytes of females with a healthy pregnancy (lane 1) and females with GDM (lanes 2 and 3). (B) TLR4 protein levels were increased in females with GDM compared with the normal controls. Data are presented as the mean ± standard deviation. ^*^P<0.05, vs. normal controls. TLR4, toll-like receptor 4; GDM, gestational diabetes mellitus.

TLR4 protein expression was observed in all monocyte samples (Fig. 2A). TLR4 protein expression levels were significantly increased in the GDM group compared with the normal control group (P<0.05; Fig. 2B).

### Serum TNF-α levels

**Figure 3:**
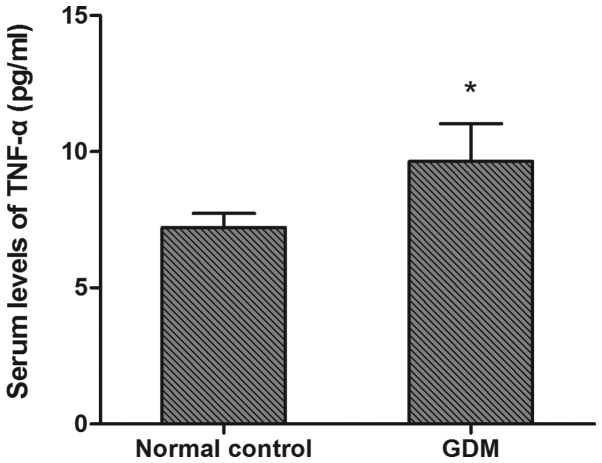
Serum levels of TNF-α in normal controls and females with GDM. Data are presented as the mean ± standard deviation. Serum levels of TNF-α were increased in females with GDM compared with the normal controls. ^*^P<0.05, vs. normal controls. TNF-α, tumor necrosis factor-α; DM, gestational diabetes mellitus.

Significantly elevated serum TNF-α levels were observed in the GDM group (9.50±1.73 pg/ml) compared with the control group (7.27±0.45 pg/ml) (P<0.05, Fig. 3).

### Correlation between TLR4 expression and serum TNF-α levels

**Figure 4:**
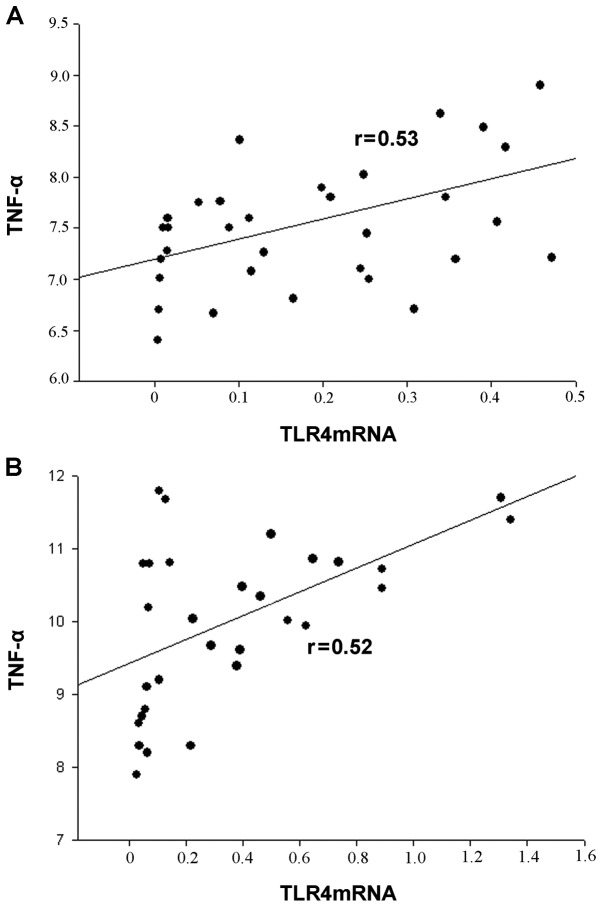
Correlation between TLR4 mRNA expression and TNF-α. (A) Normal control group; (B) females with GDM. There was a positive correlation between the TLR4 mRNA expression in maternal monocytes and the serum concentrations of TNF-α in the normal controls and females with GDM. TLR4, toll-like receptor 4; TNF-α, tumor necrosis factor-α; GDM, gestational diabetes mellitus.

TLR4 mRNA levels correlated with serum TNF-α levels in all participants. There was a positive correlation between the serum TNF-α levels and TLR4 mRNA expression in monocytes (Fig. 4).

## Discussion

To the best of our knowledge, the present is the first to compare the levels of TLR4 gene expression between females with GDM and healthy pregnant females. The results showed that TLR4 gene expression levels were significantly increased in patients with GDM compared with healthy pregnant females. Serum TNF-α levels were higher in the GDM group than in the normal control group. In addition, a positive correlation was also observed between TLR4 mRNA expression and serum TNF-α levels in this study.

Inflammation is characterized by increased levels of circulating biomarkers of inflammation and monocyte activity. The relationships between inflammation, hyperglycemia and IR have been widely studied in patients with diabetes and have been hypothesized to be mediated via the activation of the innate immune system. TLR4 is an important mediator in the innate and cytokine-activated immune systems. In addition, TLR4 is required for the adaptive immune response, and its activation mediates a signaling pathway involved in the transcriptional expression of proinflammatory cytokines and chemokines (14). TLR4 has also been identified as the primary receptor for LPS and is expressed in numerous types of cells. TLR4 gene expression and activation have been demonstrated to be increased in the monocytes of patients with hyperglycemia (15). In addition, upregulated TLR4 expression is likely to lead to disease-resistant effects, and TLR4 has a proinflammatory role in the occurrence of diabetes and its complications (11,16).

It has been suggested that GDM is a state of chronic inflammation (17). In DM, the aggravated inflammation may be mediated by TLRs via the activation of the innate immune pathway (18). TLR4 activation and downstream cytokine production may lead to the development of diabetes (19). However, the underlying mechanisms remain unknown. Increased TLR4 expression has been observed to correlate with enhanced nuclear factor (NF)-κB activation in response to the TLR4 ligand, resulting in elevated levels of proinflammatory cytokines (19). NF-κB is a well-recognized transcription factor that regulates the production of proinflammatory cytokines, including TNF-α and interleukin 1 (IL-1) (20). TLR4 also activates the mitogen-activated protein kinase (MAPK) signaling pathway, leading to the increased transcription of genes involved in inflammation (21). In addition, TLR4 activation affects chemokine (C-X-C motif) ligand 10, inducing apoptosis of islet β cells (22). These observations suggest that TLR4 may lead to the activation of various downstream pathways and cause diverse pathophysiological events. These events may induce the development of GDM.

GDM is characterized by decreased maternal insulin sensitivity or increased IR. In a previous study, the majority of females with GDM appeared to exhibit β-cell dysfunction under a background of chronic IR (23). IR was revealed to be associated with the innate immune response (24). A recent study suggested that TLR4 and downstream pathways (MAPK and NF-κB) are important in the pathogenesis of IR (25). In addition, abnormal TLR4 expression may induce an inflammatory response in insulin-resistant subjects (26). These results indicate that TLR4 expression in females with GDM may result in IR. In patients with GDM, IR may lead to hyperglycemia, fetal macrosomia, a higher likelihood of developing obstetric complications and a higher risk of stillbirth (27).

The function of the TLR4 gene has been widely studied in immune system cells, including monocytes and macrophages. Monocytes effectively regulate specific and non-specific immunological responses, which avoid damage to the organisms by pathogens. Thus, phagocytosis by monocytes is important in the innate immune response. Monocytes phagocytose a portion of debris left from the digestion of a pathogen and present it as an antigen to the adaptive immune system (28). The upregulation of TLR4 expression has been demonstrated to promote the phagocytic capacity of monocytes (29). Hence, TLR4 is expressed in monocytes and TLR4 signaling is necessary for phagocytosis by monocytes.

TNF-α is a proinflammatory cytokine secreted predominantly by monocytes and macrophages. The results of the present study are consistent with a previous observation that serum TNF-α levels were increased in females with GDM compared with healthy pregnant females (30). Therefore, hyperglycemia-induced TNF-α release in patients with GDM may contribute to the underlying pathogenesis of GDM. It has been suggested that the production of TNF-α is induced by the activation of TLR4. The activation of TLR4 has been shown to lead to the activation of NF-kB, which results in the production of TNF-α (31). In addition, the activation of monocytes, induced by the TLR4-mediated c-Jun N-terminal kinase signaling, may also cause the secretion of TNF-α (32). These results suggest that the activation of TLR4 is associated with the upregulation of TNF-α. Therefore, the elevated expression of TLR4 is able increase serum TNF-α level. An increase in serum TNF-α levels from early to late pregnancy was correlated with a decrease in insulin sensitivity (33), which suggested that TNF-α is associated with the development of IR. TNF-α is a significant predictor of IR in patients with GDM through its ability to decrease the tyrosine kinase activity of the insulin receptor (27).

In conclusion, the novel observations of the present study indicate that TLR4 expression increases in monocytes in GDM and a positive correlation exists between TLR4 mRNA expression in monocytes and serum TNF-α level in females with GDM. These results suggest that a selective interference with TLR4 may present an opportunity for the treatment of IR and GDM.

## Figures and Tables

**Table I:** Demographic data in the GDM and normal control groups.

Characteristics	Normal control (n=32)	GDM (n=30)	P-value
Age, years	29.6±3.3	30.9±4.7	NS
Gestational age, days	272.6±4.5	273.9±5.6	NS
Fetal weight, g	3,230.0±304.7	3,373.0±418.4	NS
Systolic blood pressure, mmHg	114.5±14.7	108.6±8.3	NS
Diastolic blood pressure, mmHg	72.9±8.7	72.5±6.8	NS
BMI, kg/m^2^	22.1±1.9	23.3±2.8	NS
Fasting glucose, mmol/l	4.1±0.5	5.8±0.8^a^	<0.05
Serum fasting insulin, μU/ml	8.6±4.2	14.5±10.2^a^	<0.05
HOMA-IR	1.58±0.7	4.6.0±2.2^a^	<0.05

Results are presented as the mean ± standard deviation.

a P<0.05 was set as statistically significant.

GDM, gestational diabetes mellitus; NS, not significant; BMI, body mass index; HOMA-IR, homoeostasis model assessment-insulin resistance.
